# RiseTx: testing the feasibility of a web application for reducing sedentary behavior among prostate cancer survivors receiving androgen deprivation therapy

**DOI:** 10.1186/s12966-018-0686-0

**Published:** 2018-06-07

**Authors:** Linda Trinh, Kelly P. Arbour-Nicitopoulos, Catherine M. Sabiston, Scott R. Berry, Andrew Loblaw, Shabbir M. H. Alibhai, Jennifer M. Jones, Guy E. Faulkner

**Affiliations:** 1 Faculty of Kinesiology and Physical Education, University of Toronto, Toronto, ON Canada; 2 Sunnybrook Odette Cancer Centre, Toronto, ON Canada; 3 Department of Medicine, University Health Network & University of Toronto, Toronto, ON Canada; 4 Cancer Survivorship Program, Princess Margaret Cancer Centre, Toronto, ON Canada; 5 School of Kinesiology, University of British Columbia, Vancouver, BC Canada

**Keywords:** Sedentary behavior, Prostate cancer, Feasibility, web-based, physical activity

## Abstract

**Background:**

Given the high levels of sedentary time and treatment-related side effects in prostate cancer survivors (PCS), interventions targeting sedentary behavior (SED) may be more sustainable compared to physical activity (PA).

**Purpose:**

To examine the feasibility of a web-based intervention (RiseTx) for reducing SED and increasing moderate-to-vigorous physical activity (MVPA) among PCS undergoing ADT. Secondary outcomes include changes in SED, MVPA, light intensity PA, and quality of life.

**Methods:**

Forty-six PCS were recruited from two cancer centres in Toronto, Ontario, Canada between July 2015–October 2016. PCS were given an activity tracker (Jawbone), access to the RiseTx website program, and provided with a goal of increasing walking by 3000 daily steps above baseline levels over a 12-week period. A range of support tools were progressively released to reduce SED time (e.g., self-monitoring of steps) during the five-phase program. Objective measures of SED, MVPA, and daily steps were compared across the 12-week intervention using linear mixed models.

**Results:**

Of the 46 PCS enrolled in the study, 42 completed the SED intervention, representing a 9% attrition rate. Measurement completion rates were 97 and 65% at immediately post-intervention and 12-week follow-up for all measures, respectively. Overall adherence was 64% for total number of logins (i.e., > 3 visits each week). Sample mean age was 73.2 ± 7.3 years, mean BMI was 28.0 ± 3.0 kg/m^2^, mean number of months since diagnosis was 93.6 ± 71.2, and 72% had ADT administered continuously. Significant reductions of 455.4 weekly minutes of SED time were observed at post-intervention (*p* = .005). Significant increases of + 44.1 for weekly minutes of MVPA was observed at immediately post-intervention (*p* = .010). There were significant increases in step counts of + 1535 steps from baseline to post-intervention (*p* < .001).

**Conclusions:**

RiseTx was successful in reducing SED and increasing MVPA in PCS. PCS were satisfied with the intervention and its components. Additional strategies may be needed though for maintenance of behavior change. The next step for RiseTx is to replicate these findings in a larger, randomized controlled trial that will have the potential for reducing sedentary time among PCS.

**Trial registration:**

NCT03321149 (ClinicalTrials.gov Identifier).

## Background

Advances in prostate cancer treatment, particularly through the use of androgen deprivation therapy (ADT), have contributed to the growing survival rate [1, 2]. Although ADT improves survival from prostate cancer, it is associated with many adverse health effects such as decreased strength, impaired physical function, and physical inactivity [3]. As such, supportive care interventions are needed to reduce the chronic and late-appearing effects during the transition into survivorship.

Physical activity (PA) has a positive impact on clinical outcomes such as improvement in overall quality of life (QoL), cancer-specific mortality, and reducing treatment-related toxicities in cancer survivors [4], including PCS [5–7]. Despite these benefits of regular PA, the majority of PCS are not meeting public health PA guidelines [8–11]. There is also a significant decrease in PA levels during adjuvant therapy and levels remain low post-treatment [12]. Short-term supervised PA programs can improve fitness and patient-reported outcomes in PCS [13], but PA declines significantly after the intervention and long-term adherence is often difficult [13–15].

Therefore, alternative approaches to alleviating the burden of ADT among PCS are needed. Many of these chronic health conditions may be preventable with lifestyle changes such as reduced sedentary behavior (SED) – yet there are no known effective strategies aimed at reducing SED among men on ADT. SED is defined as any waking behavior characterized by a low energy expenditure (i.e., ≤1.5 resting metabolic equivalents) while in a sitting or reclining posture [16]. The focus on SED is of practical and clinical value and may be a more feasible targeted intervention approach. There are no reported interventions targeting SED in cancer survivors. Furthermore, few studies have examined relationships among SED, treatment side effects, and QoL in this population [11, 17].

Targeting SED makes intuitive sense given that the majority of cancer survivors’ time is spent sedentary. Independent of PA, high volumes of SED are associated with chronic disease-related risk factors such as central adiposity, elevated blood glucose and insulin [18]. These factors may be amplified through the course of ADT [17–19]. Therefore, an important complementary focus is to reduce time spent sedentary in addition to increasing habitual PA.

The Medical Research Council (MRC) advises a systematic approach to intervention development, which includes an iterative process of identification of the evidence base; developing a theoretical understanding, and using pilot work to inform final modifications to the intervention prior to evaluation [20]. The framework of RiseTx follows this iterative process by using a two-phase feasibility study where Phase 1 involved formative research to tailor the final content and structure of the intervention, while Phase 2 was to test the feasibility of a web-based intervention for decreasing SED. In Phase 1, focus groups were conducted with PCS on ADT to address perceptions regarding SED and preferences for a web-based SED intervention. Many PCS expressed that the design should be easy to use; have an alerting function to interrupt sitting; have the ability to track and monitor PA levels; be tailored to the individual; and involve social support [21]. The findings of Phase 1 informed the development of the 12-week web-based SED intervention, Reducing Sitting Everyday for Treatment (RiseTx), for men on ADT. To the best of our knowledge, this is the first intervention to target SED in cancer survivors.

Thus, the purpose of this study was to examine feasibility indicators such as recruitment, adherence, number of adverse events, and acceptability of the RiseTx intervention in PCS on ADT. Secondary outcomes were changes in SED, MVPA, light-intensity PA, and QoL.

## Methods

### Recruitment and eligibility criteria

Men who were diagnosed with prostate cancer and prescribed ADT were recruited predominantly through Genitourinary (GU) clinics in two large cancer centers in Toronto, Ontario, Canada between July 2015 and October 2016. Inclusion criteria included: i) ≥ 18 years of age; ii) men with localized or asymptomatic metastatic primary prostate cancer (Stage I-III); iii) currently receiving ADT (continuous and/or intermittent) for at least 6 months; iv) active e-mail address to access the intervention website; v) proficient in English; vi) insufficiently active (< 150 min of moderate-intensity PA/week); vii) no uncontrolled co-morbidities; and viii) medical clearance from the primary healthcare provider. The study was approved by the research ethics board. All participants provided written informed consent.

### Sample size calculation

An a priori sample size calculation was not conducted due to the feasibility nature of this study [22]. A post hoc calculation with 46 participants revealed 90% power to detect a medium effect size of 0.30 (Cohen’s *f*) for our primary (i.e., feasibility) and secondary outcomes (i.e., PA, SED, QoL) using a 2-tailed test with α = 0.05 [23]. Given that this was a feasibility study with a small sample size, the results were interpreted for both statistical and clinical significance based on the guidelines for minimal important differences (MID) on the QoL scales [24].

#### Study design

The feasibility study was a prospective, single-arm design. Interested participants completed a baseline questionnaire and were immediately provided with access to the RiseTx web-based intervention.

#### The RiseTx intervention

**Fig. 1:**
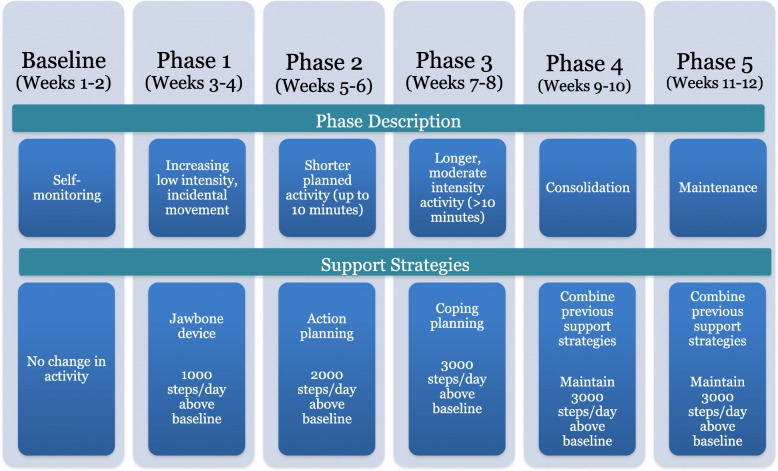
RiseTx Application Phases

**Fig. 2:**
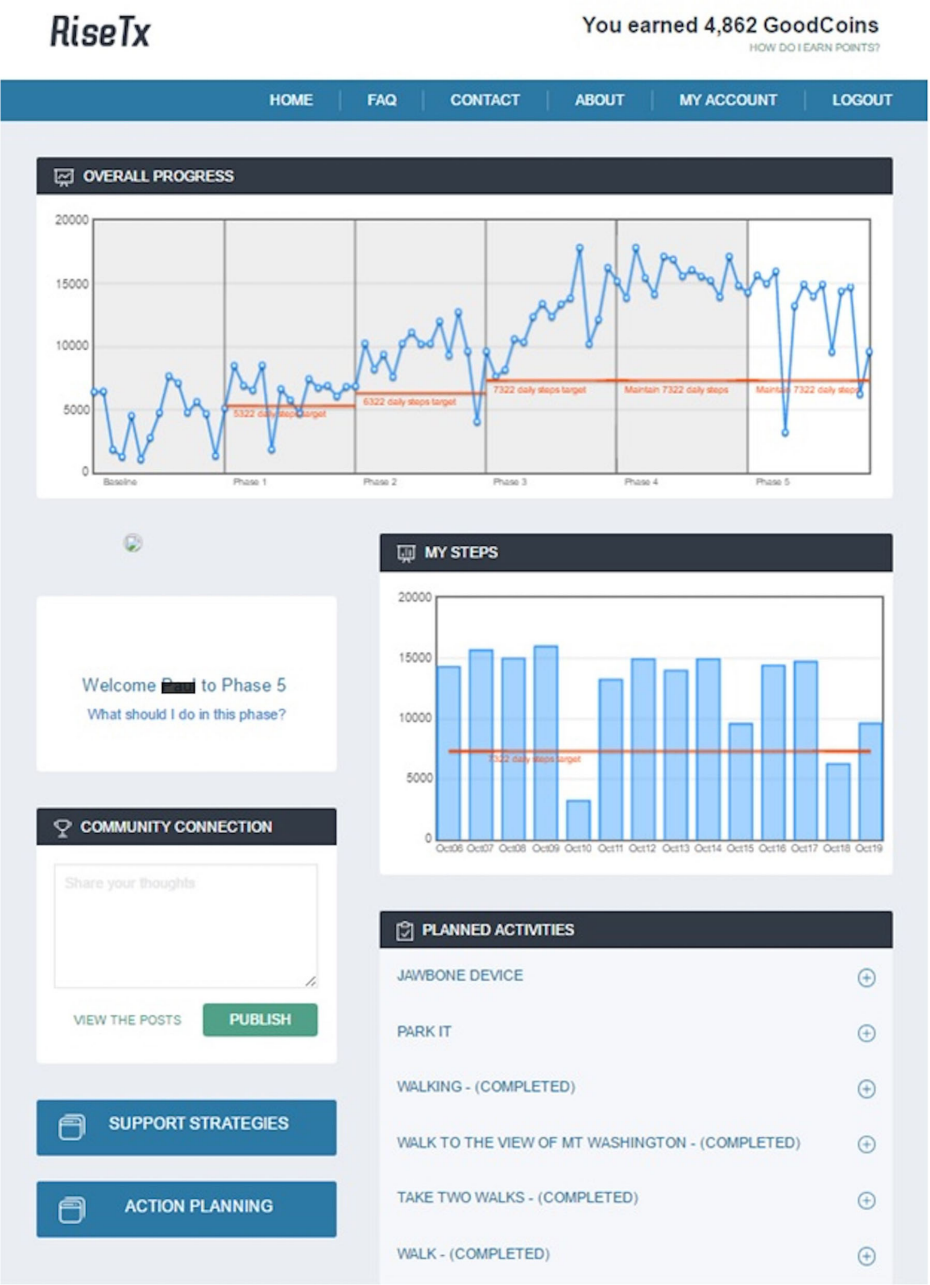
Sample Homepage from the RiseTx Web-based Application

In the first 10 days following recruitment, participants met with the research coordinator and were provided with an accelerometer (GTX3, ActiGraph, Pensacola, FL, USA) and completed self-report baseline measures. Provided along with the RiseTx intervention is the Jawbone UP 24 (Jawbone, San Francisco, CA, USA), which is a wrist-worn activity tracker that can assess activity patterns throughout the day and provide sensory alerts to stand after prolonged sitting (i.e., ≥30 min of sedentary time). The intervention consisted of five phases following initial data collection, including a baseline phase (weeks 1–2; see Fig. 1). Phases I-III (weeks 3–6) involved progressive release of self-regulatory strategies (e.g., action planning) and targeted changes in both sitting time (and breaks in sitting time) and step counts. A 4-week consolidation phase (Phase IV and V; weeks 9–12) followed, where participants received weekly reminders that encouraged them to continue to use the RiseTx application to practice combining the different self-regulatory strategies learned in Phases I-III. Based on a previously tested ramped step count approach that focuses on increasing walking by an extra 1000 daily steps over a set period [25], participants attempted to increase daily steps by an additional + 1000 daily step increment set above the previous phase (see Fig. 2).

Incentives were built into the intervention to increase engagement with the RiseTx application [26] such as logging in to the website every day and reaching the step goal target for a particular phase. Rewards were accumulated in the form of points that could be redeemed for a variety of items and/or donating them to a charity of choice (maximum $50 CAD). Following the intervention, there was a 12-week maintenance period (weeks 13–24) where participants no longer received weekly self-regulatory practice reminders, yet still had access to the application.

### Measures

#### Feasibility measures

Feasibility was determined through rates of recruitment, measurement completion, attrition, intervention adherence (i.e., tracked through website analytics such as number of logins), number of adverse events, and intervention evaluation items assessing burden and satisfaction. Acceptability was measured through an intervention satisfaction survey completed at post-intervention assessing perceptions and overall impressions of the RiseTx intervention. A predefined success criteria was implemented to judge the feasibility of the RiseTx intervention which was based on previous research [27–29] and included the following: 1) study recruitment rates (~ 10 participants recruited per month over a 6-month period); 2) intervention adherence (≥ 3 visits by participants each week to the RiseTx platform; 3) measurement completion rates (≥75% complete baseline, post-intervention, and follow-up measures); 4) attrition rates (≤20% drop-out rate); 5) acceptability (> 75% rate their participation as satisfactory or very satisfactory), 6) number of adverse events, and 7) intervention evaluation items assessing burden and satisfaction. Acceptability was measured through an intervention satisfaction survey completed at post-intervention assessing perceptions and overall impressions of the RiseTx intervention.

### Secondary outcomes

All measures were completed at baseline (T0), post-intervention (i.e., 12 weeks; T1) and follow-up (i.e., 24 weeks; T2). Standard demographic and medical variables were also collected at baseline.

#### Physical activity and sedentary behavior

Change in volume of SED and PA were measured by ActiGraph Model GT3X accelerometers (Actigraph, Pensacola, Florida). A bout is defined as any continuous period of SED time with the bout stopped when > 100 counts for an epoch are recorded. A break is defined as any 5-s epoch change from SED to light-intensity PA or greater. Participants were asked to wear the accelerometer for seven consecutive days during waking hours, except for periods of bathing/showering or other water-based activities. Data were downloaded in 60-s epochs and converted to mean counts per minute to estimate daily minutes of light (101–1951 counts•minute^− 1^), moderate (1952–5724 counts•minute^− 1^), and vigorous (> 5725 counts•minute^− 1^) PA based on established cut-points [27], while controlling for the number of days the accelerometer was worn. Data were analyzed if there were no extreme counts (> 20,000) and if data were available for at least 600 min on 4 or more days per assessment period.

Weekly step counts were collected using the Jawbone UP24 (Aliphcom, San Francisco, CA) activity tracker. The monitor has a rechargeable battery and syncs to a smartphone or tablet via an Internet connection or Bluetooth to upload step counts to the RiseTx website.

#### Quality of life

QoL was assessed by the validated Functional Assessment of Cancer Therapy-General (FACT-G) which consists of physical well-being (PWB), functional well-being (FWB), emotional well-being (EWB), and social well-being (SWB) [30]. The FACT-Fatigue (FACT-F) scale included the 27 items from the FACT-General (FACT-G) scale plus the 13 item fatigue subscale [30, 31]. The PWB, FWB, and fatigue scales were summed to form the Trial Outcome Index-Fatigue (TOI-F) [30, 31]. We also included the validated FACT-Prostate (FACT-P) subscale which contains 12 questions that assess the most important targeted symptoms and concerns for participants [32]. On all scales, higher scores indicate better QoL and less symptoms.

### Demographic and medical information

The demographic variables included: age, marital status, highest level of education, current employment status, ethnicity, and height and weight to calculate body mass index (BMI). The medical variables included: time since diagnosis, disease stage, ADT administration method, current/prior treatments, previous recurrence, and current disease status, which have been used previously in studies with cancer survivors [21].

### Statistical analysis

All statistical analyses were performed using SPSS 23 (SPSS Inc., Chicago, IL). Descriptive statistics were calculated to describe the sample population. Linear mixed models were used to model each outcome measure (i.e., PA, SED, QoL) at the three time points [baseline (T0), post-intervention (i.e., 12 weeks; T1) and follow-up (i.e., 24 weeks; T2)]. Our analysis was adjusted for accelerometer wear time and baseline MVPA. For these analyses, all enrolled participants were retained regardless of missing data or incomplete participation. All statistical tests were two-sided.

## Results

**Fig. 3:**
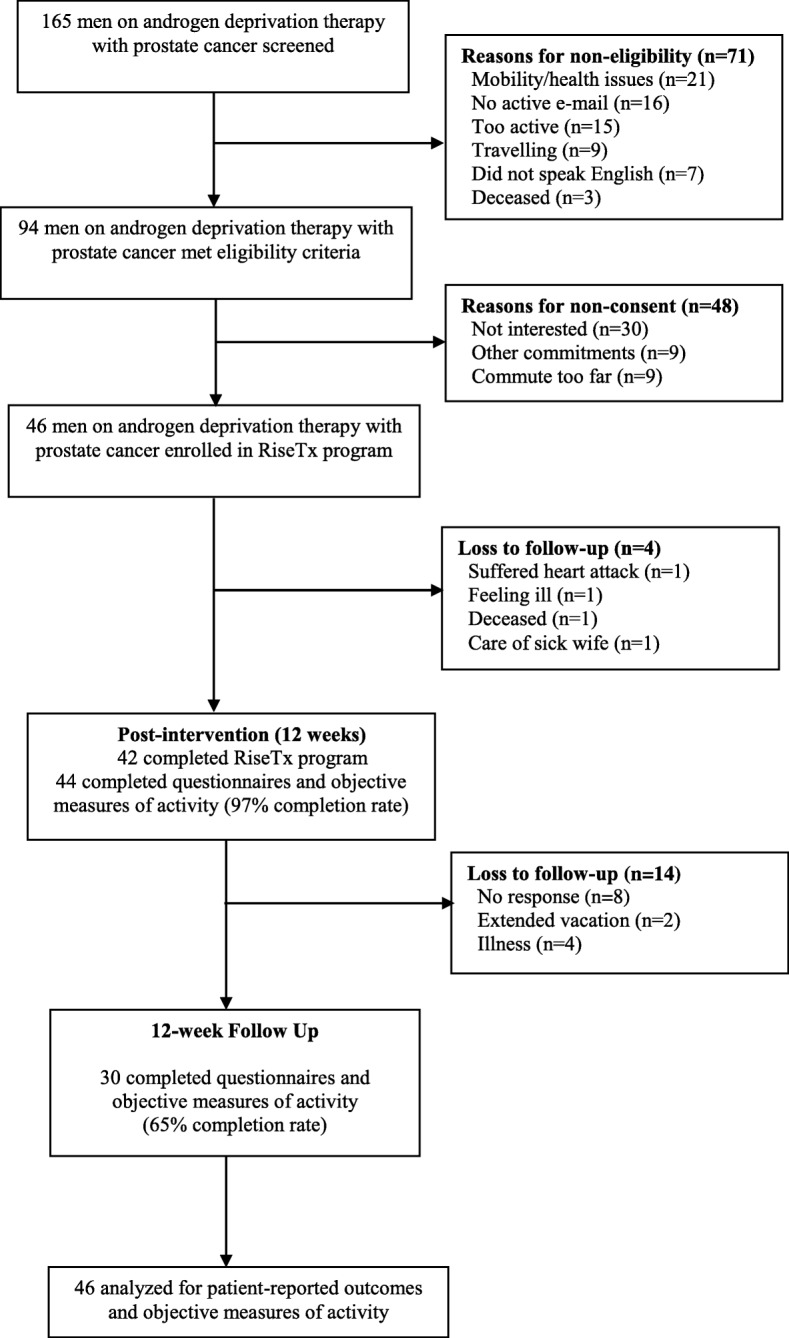
Flow of Participants through the Study

The flow of participants through the study is reported in Fig. 3.

### Feasibility outcomes

Briefly, 165 participants were screened for eligibility, of whom 94 met eligibility criteria. Of the 94 participants that met the eligibility criteria, 46 participants were interested, generating a 49% response rate (46/94). On average, 3 participants per month were recruited during the study recruitment period. Of the 46 participants enrolled in the study, 42 completed the SED intervention, representing a 9% attrition rate or, conversely, a 91% retention rate. Measurement completion rates for self-reported outcomes were 97 and 65% at post-intervention and 12-week follow-up, respectively.

The overall adherence rate to the intervention was 72% (33/46) for total number of logins (i.e., > 3 visits each week). In terms of step counts, 59% of the 46 participants (*n* = 27) met the target step count for Phase 1 (i.e., + 1000 steps above baseline), 46% met the target step count for Phase 2 (*n* = 21) (i.e., + 2000 steps above baseline), 39% met the target step count for Phase 3 (*n* = 18) (i.e., + 3000 steps above baseline), and 35 and 26% of participants met the target step count for Phase 4 (*n* = 16) and 5 (*n* = 12) (i.e., maintain + 3000 steps above baseline) respectively.

Two participants experienced adverse events with one patient suffering a myocardial infarction and one patient deceased for unknown causes during the intervention. However, it is unknown whether the two adverse events were related to the SED intervention as both participants were provided with physician clearance and had no previous cardiac history before the start of the study.

### Participant characteristics

Baseline demographic and medical characteristics, as well as behavioral characteristics of participants are reported in Tables 1 and 2, respectively. Overall, participants had a mean age of nearly 74 years, 84.8% were married, and the mean BMI was 28 kg/m^2^. The mean number of months since diagnosis was 93.6 months, 54.3% had received surgery, 63.0% had ADT administered continuously, and 65.2% reported localized prostate cancer. Participants spent 73.0% of their total time sedentary (525.9 min/day), 25.2% in light-intensity PA (192.5 min/day), and 1.8% in MVPA (14.3 min/day).

**Table 1:** Demographic and medical characteristics of men with prostate cancer on androgen deprivation therapy in Toronto, Ontario, Canada, July 2015–October 2016 (*N* = 46)

Variable	Mean ± SD or n (%)
Age	73.2 ± 7.3
Marital Status	
Never married	3 (6.5)
Married/common law	39 (84.8)
Widowed	2 (4.3)
Divorced	2 (4.3)
Education	
Some high school	4 (8.7)
Completed high school	4 (8.7)
Some university/college	13 (28.3)
Completed university/college	16 (34.8)
Some/completed graduate school	9 (19.5)
Employment status	
Employed full−/part-time	11 (23.9)
Retired	34 (73.9)
Other	1 (2.2)
Ethnicity	
White	37 (80.4)
Black	4 (8.7)
South Asian	2 (4.3)
Southeast Asian	1 (2.2)
Other	2 (4.3)
Body mass index	28.0 ± 3.0
Healthy weight	8 (17.4)
Overweight	26 (56.5)
Obese	12 (26.1)
Number of comorbidities	
None	4 (8.7)
1	11 (23.9)
2	14 (30.4)
≥ 3	18 (36.9)
^a^Most common comorbidities	
High blood pressure	27 (61.4)
High cholesterol	19 (43.2)
Arthritis	15 (34.1)
Smoking status	
Never smoked	23 (50.0)
Ex-smoker	22 (47.8)
Regular smoker	1 (2.2)
Alcohol consumption	
Never	8 (17.4)
Less than once a month	7 (15.2)
2–3 times a month	5 (10.9)
Once a week	4 (8.7)
2–3 times a week	6 (13.0)
4–6 times a week	8 (17.4)
Every day	8 (17.4)
Months since diagnosis	93.6 ± 71.2
Disease stage	
Localized	30 (65.2)
Metastatic	15 (32.6)
Unsure	1 (2.2)
Androgen deprivation therapy administration	
Continuous	29 (63.0)
Intermittent	15 (32.6)
Unsure	2 (4.3)
^a^Current/prior prostate cancer treatment	
Surgery	25 (54.3)
Radiation	38 (82.6)
Chemotherapy	3 (6.5)
Current treatment status	
Completed treatment	19 (41.3)
Receiving treatment	27 (58.7)
Cancer disease recurrence	
Yes	16 (34.8)
No	30 (65.2)
Current cancer disease status	
Disease-free	15 (32.6)
Existing disease	31 (67.4)

^a^ could check more than one

**Table 2:** Behavioral and quality of life characteristics of men with prostate cancer on androgen deprivation therapy in Toronto, Ontario, Canada, July 2015–October 2016 (*N* = 46)

Variable	M ± SD
Objectively-assessed physical activity (*N* = 45)	
Average weekly light-intensity minutes	1239.2 ± 465.7
Average light-intensity minutes per day	192.5 ± 62.4
Percent time in light-intensity PA (%)	25.2 ± 6.9
Average weekly MVPA minutes	93.1 ± 89.4
Average MVPA minutes per day	14.3 ± 13.4
Percent time in MVPA (%)	1.8 ± 1.6
Objectively-assessed sedentary time (*N* = 45)	
Average weekly minutes	3514.4 ± 718.6
Average minutes per day	525.9 ± 84.6
Percent time in sedentary behavior (%)	73.0 ± 7.6
Quality of life	
Physical well-being (0–28)	23.5 ± 3.8
Functioning well-being (0–28)	19.7 ± 5.4
Emotional well-being (0–28)	17.9 ± 5.2
Social well-being (0–24)	20.1 ± 4.7
FACT-General (0–108)	82.0 ± 13.4
FACT-Fatigue (0–160)	122.1 ± 19.8
FACT-Prostate (0–156)	114.9 ± 18.1
Trial outcome index-Fatigue (0–108)	84.5 ± 14.4

*FACT* Functional Assessment of Cancer Therapy, *PA* physical activity, *MVPA* moderate-to-vigorous physical activity

### Physical activity and sedentary behavior

Table 3 provides the change in SED and PA at baseline to post-intervention, and from baseline to 12-week follow-up. A significant reduction of 455.4 weekly minutes of sedentary time was observed at post-intervention [95% CI: -766.6 to 144.2; *p* = .005]. No significant effects were found for weekly minutes of sedentary time from baseline to 12-week follow-up. A significant increase of + 44.1 for weekly minutes of MVPA was observed at post-intervention [95% CI: 11.1 to 77.0; *p* = .010], but no significant effects were found at 12-week follow-up. There were no significant effects found for light-intensity PA from baseline to post-intervention and at 12-week follow-up.

**Table 3:** Effects of a sedentary behavior intervention on activity levels in men with prostate cancer on androgen deprivation therapy in Toronto, Ontario, Canada, July 2015–October 2016 (*N* = 46)

	Baseline (T0)	Post-intervention (T1)	12-week Follow-Up (T2)	^a^Adjusted Difference in Mean Change (T0-T1)		^a^Adjusted Difference in Mean Change (T0-T2)	
Outcome	Mean (SE)	Mean (SE)	Mean (SE)	Mean [95% CI]	*p*	Mean [95% CI]	*p*
Total SED minutes	3514.4 (105.7)	3058.9 (187.9)	3272.0 (157.2)	-455.4 [-766.6 to -144.2]	.005	− 242.4 [− 565.2 to 80.5]	.14
Total time spent in SED bouts of ≥30 min	1224.7 (65.9)	1078.0 (84.3)	1166.4 (88.6)	− 148.7 [− 315.2 to 17.7]	.079	−58.3 [− 262.1 to 145.5]	.57
Total # of breaks in time spent in SED bouts of ≥30 min	25.9 (1.4)	22.8 (1.8)	25.0 (1.9)	−3.1 [− 6.4 to 0.4]	.078	− 0.92 [− 5.1 to 3.3]	.66
Total Light PA minutes	1239.2 (70.0)	1148.2 (91.5)	1210.5 (80.1)	−91.0 [− 236.4 to 54.4]	.22	− 28.6 [− 198.2 to 141.0]	.74
Total MVPA minutes	93.1 (14.5)	137.1 (22.8)	122.1 (23.9)	+ 44.1 [11.1 to 77.0]	.010	+ 29.0 [− 14.2 to 72.2]	.18
Total # of MVPA bouts of ≥10 min	4.3 (3.1)	3.1 (0.8)	2.4 (0.7)	−1.2 [− 7.1 to 4.6]	.67	− 1.9 [− 8.1 to 4.4]	.55

Total SED minutes: the total number of minutes spent in sedentary activity (defined as all minutes with an average activity count of < 100 counts•minute-1)

Total time spent in SED bouts of ≥30 min: the total number of minutes spent in sedentary activity in a sedentary bout lasting ≥30 min. A sedentary bout is defined as ≥1 consecutive minutes with < 100 counts•minute-1

Total # of breaks in time spent in SED bouts of ≥30 min: the total number of interruptions (i.e., accelerometer counts per minute were ≥ 100) in sedentary time lasting ≥1 min in a sedentary bout lasting ≥30 min

Total # of MVPA bouts of ≥10 min: the total number of bouts spent in MVPA lasting ≥10 min. A MVPA bout is defined as ≥10 consecutive minutes with ≥1952 counts•minute-1

*SED* sedentary behavior, *MVPA* moderate-to-vigorous physical activity

^a^Difference in mean change adjusted for accelerometer wear time and baseline MVPA

### Step counts

**Fig. 4:**
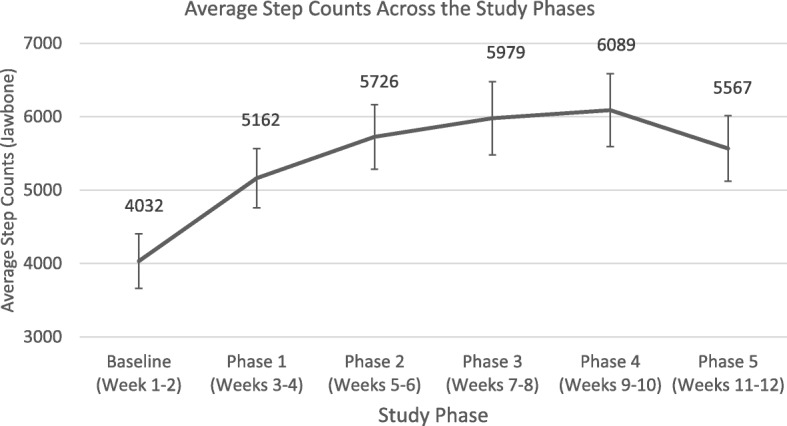
Average Step Counts Across the Study Phases Measured by the Jawbone UP24 Activity Tracker (*N* = 46)

Figure 4 illustrates the step counts accumulated throughout baseline and Phases I-V of the intervention. There were significant increases in step counts of + 1535 steps from baseline to post-intervention (*p* < .001).

### Quality of life

Table 4 present changes in general QoL and cancer-specific QoL at baseline to post-intervention and at baseline to 12-week follow-up. There were significant improvements in EWB where there was a + 1.2 point difference at post-intervention (95% CI: 0.2–2.1; *p* = .018), but not at 12-week follow-up. However, this was not meaningful based on guidelines for minimally important differences (MID). No significant effects were observed at post-intervention and 12-week follow-up for the FACT subscales or other cancer-specific QoL measures.

**Table 4:** Effects of a Sedentary Behavior Intervention on Quality of Life in Men With Prostate Cancer on Androgen Deprivation Therapy in Toronto, Ontario, Canada, July 2015–October 2016 (*N* = 46)

	Baseline (T0)	Post-intervention (T1)	12-week Follow-Up (T2)	^a^Adjusted Difference in Mean Change (T0-T1)		^a^Adjusted Difference in Mean Change (T0-T2)	
Outcome	Mean (SE)	Mean (SE)	Mean (SE)	Mean [95% CI]	*p*	Mean [95% CI]	*p*
Physical Well-Being (0–28)	23.5 (0.6)	23.3 (0.6)	22.7 (0.8)	−0.3 [−1.2 to 0.8]	.59	−0.8 [−2.3 to 0.6]	.27
Social Well-Being (0–28)	19.7 (0.8)	19.7 (0.7)	19.2 (0.8)	−0.0 [−1.6 to 1.6]	.99	−0.6 [−2.6 to 1.5]	.57
Emotional Well-Being (0–24)	17.9 (0.8)	19.0 (0.7)	19.0 (0.7)	+ 1.2 [0.2 to 2.1]	.018	+ 1.1 [−0.2 to 2.4]	.091
Functional Well-Being (0–28)	20.9 (0.7)	20.0 (0.8)	19.8 (0.8)	−0.8 [−2.0 to 0.3]	.15	−1.1 [−2.7 to 0.5]	.17
FACT-General (0–108)	82.0 (2.0)	82.0 (2.1)	80.5 (2.2)	+ 0.0 [−2.6 to 2.6]	.99	−1.6 [−5.3 to 2.2]	.41
FACT-Fatigue (0–160)	122.1 (2.9)	121.4 (3.4)	118.7 (3.5)	−0.6 [−4.4 to 2.9]	.68	−3.4 [−8.7 to 1.9]	.20
FACT-Prostate (0–156)	114.9 (2.7)	113.4 (3.0)	112.7 (3.2)	−1.5 [−4.7 to 1.6]	.33	−2.2 [−6.9 to 2.4]	.34
TOI-Fatigue (0–108)	84.5 (2.1)	82.6 (2.6)	80.7 (2.8)	−1.9 [−4.9 to 1.1]	.21	−3.8 [−8.2 to −0.6]	.086

Minimal important differences (MID) points for PWB (2–3); EWB (2–3); SWB (2–3); FWB (2–3); FACT-G (3–7); FACT-F (7); TOI-F (5); FACT-P (6–10)

*FACT* Functional Assessment of Cancer Therapy, *FACT-G* Functional Assessment of Cancer Therapy-General, *FACT-F* Functional Assessment of Cancer Therapy-Fatigue, *TOI-F* Trial Outcome Index-Fatigue, *FKSI-15* Kidney symptom index, *SPA + EC* Supervised physical activity plus exercise counseling, *SPA + BC* Supervised physical activity plus behavioral counseling

^a^Difference in mean change adjusted for baseline value

## Discussion

To our knowledge, the RiseTx intervention is the first study to pilot a SED intervention in PCS. The intervention met some feasibility indicators, but lacked sustained engagement as indicated by the overall adherence rate. However, the intervention had high satisfaction and low participant burden. Preliminary efficacy data suggest that there was a significant reduction of weekly minutes of sedentary time and increase in weekly minutes of MVPA observed at post-intervention. In addition, there were significant increases in step counts from baseline to post-intervention. However, few significant findings were observed at post-intervention and 12-week follow-up for the QoL measures.

While there are evidence-based interventions to increase MVPA in cancer survivors, much less is known about how to reduce SED which makes comparisons with other studies scant. The recruitment rate of 49% in our study was higher than previous web-based interventions targeting cancer survivors that includes PCS [33]. Our study generated a higher recruitment rate possibly due to our intervention targeting SED, while Forbes et al. [33] focused on a web-based PA behavior change program. However, it is important to note that the recruitment rate in our study was extended over a 15-month period. This was twice as long as was anticipated to recruit the sample needed. Our study focused on men on ADT and it is unknown what the optimal timing to implement a lifestyle behavioral change during treatment. It is possible that some PCS were not ready to resume an active lifestyle or feel unwell to do so.

The attrition rate of 9% or 91% retention rate was aligned with previous digital health interventions for behavior change among cancer survivors where Goode et al. [28] reported attrition rates between 6 and 45% for web-based delivery methods, while Roberts et al. [29] reported retention rates between 31.7–100% for digital health behavior change interventions. However, these studies were web-based interventions for PA and/or combined PA and diet. SED may represent a behavior that is more achievable and sustainable compared to PA, which requires more planning and effort [34]. PCS may find a SED intervention more attractive than an intervention to increase MVPA.

Adherence to our study was consistent with a previous meta-analysis of internet-delivered interventions to increase PA in the general and chronic-diseased populations. Davies et al. [34] found the average number of logins was 3.08 per participant per week. Our adherence rate was higher compared to a previous web-based intervention targeting PA in cancer survivors. Similarly, a meta-analysis of digital health interventions targeting PA and diet in cancer survivors found website logins ranged from 1.00 to 14.75 times per week [29]. One potential reason for differences in our engagement levels was that the RiseTx website required logging into the website every day to view the number of steps taken. We also had a progressive release of support strategies and new features of the website that were released in the first 8 weeks of the intervention which may have maintained the interest. These behavior change techniques are aligned with technology-based strategies (e.g., real-time feedback, tailored information) that are important for engagement [29, 35].

In terms of our preliminary efficacy data, the RiseTx intervention was able to decrease SED by 7.6 h and 4.0 h of sedentary time at post-intervention and at 12-week follow-up, respectively. Sustained engagement is problematic for many digital health interventions with cancer survivors [28, 29, 34]. Our study had an overall adherence rate of 64% that while higher than previous studies, still represents difficulties with engagement. Our intervention integrated techniques to encourage engagement such as progressive release of support strategies throughout the phases of the intervention, reminders, user-friendly design, tailored and individualized components, and real-time feedback of step counts. Understanding the techniques that foster effective engagement throughout the intervention may be warranted.

In addition, approximately half of PCS were able to meet the target step count for the early phases of the intervention. However, during the maintenance phases of the intervention, adherence to target step counts tapered off. It may have been too challenging for PCS to ramp up to greater than 3000 steps during the maintenance phase given many of them are older and inactive PCS. Beginning the intervention with smaller increments to maintain the step counts over the course of the intervention may be more appropriate. It is interesting to note that increases in step counts were reflected in increases in MVPA. For many older PCS, increasing walking time is considered an aerobic activity where the intensity may have been at the level of MVPA rather than light-intensity PA [36]. However, these findings suggest the utility of interventions combining the focus on reducing SED and increasing PA for PCS.

Despite positive behavior change in our intervention, no clinically relevant changes were observed in many of the QoL measures. This is not surprising given the study was underpowered and many PCS had high QoL scores at baseline. Previous studies examining objectively-assessed SED in colon cancer survivors [37] and PCS [17] and self-reported SED in PCS [38], have also demonstrated no or few associations with QoL.

This is the first study to deliver a web-based intervention to reduce SED in cancer survivors and in PCS. Preliminary data demonstrated the support of a SED intervention for reducing sitting time and increasing MVPA. Two adverse events occurred during the intervention and future research should consider exercise stress testing to screen for ischemic heart disease in previously sedentary men who wish to become physically active. Although our study experienced adverse events, light-intensity PA (i.e. walking) is generally regarded as safe and feasible for older adults, including PCS [13]. The strengths of this study include the novel target in sedentary time in cancer survivors, and the application of a simple and easy-to-use web-based intervention that is scalable and has the potential to reduce sedentary time. The intervention was also informed by preferences of PCS based on formative research [21]. Moreover, the use of objective measures of sedentary time via accelerometry was beneficial for reducing recall bias. Future studies should consider using inclinometers (i.e., ActivPALs) in conjunction with accelerometers for complete data. Limitations of this study include selection bias as PCS recruited into the study had to have an active e-mail address to participate. Given the feasibility study, we were unable to determine the elements of the intervention that were most important and effective for changing sedentary time. It was unclear which combinations of the intervention components would be optimal, and this should be addressed in larger RCTs targeting SED in cancer survivors.

In conclusion, the RiseTx intervention was associated with a reduction in SED and increased MVPA among PCS suggesting the utility and feasibility of interventions that target these behaviors simultaneously. Additional strategies may be needed for maintenance of behavior change for PCS, especially to continue engagement with the website and to use the intervention components. Web-based interventions for SED are highly accessible and offer an alternative approach for cancer survivorship care. Health professionals working with cancer survivors should consider encouraging a reduction in SED as an initial step towards increasing activity levels for better health outcomes in PCS.

## Abbreviations

ADT: androgen deprivation therapy
BMI: body mass index
EWB: emotional well-being
FACT-G: Functional Assessment of Cancer Therapy-General
FACT-P: Functional Assessment of Cancer Therapy-Prostate
FWB: functional well-being
GU: genitourinary
MID: minimal important differences
MRC: Medical Research Council
MVPA: moderate-to-vigorous physical activity
PA: physical activity
PCS: prostate cancer survivors
PWB: physical well-being
QoL: quality of life
SED: sedentary behavior
SWB: social well-being
TOI-F: Trial Outcome Index-Fatigue
