# Correction to: RiseTx: testing the feasibility of a web application for reducing sedentary behavior among prostate cancer survivors receiving androgen deprivation therapy

**DOI:** 10.1186/s12966-018-0757-2

**Published:** 2018-12-04

**Authors:** Linda Trinh, Kelly P. Arbour-Nicitopoulos, Catherine M. Sabiston, Scott R. Berry, Andrew Loblaw, Shabbir M. H. Alibhai, Jennifer M. Jones, Guy E. Faulkner

**Affiliations:** 1 Faculty of Kinesiology and Physical Education, University of Toronto, Toronto, ON Canada; 2 Sunnybrook Odette Cancer Centre, Toronto, ON Canada; 3 Department of Medicine, University Health Network & University of Toronto, Toronto, ON Canada; 4 Cancer Survivorship Program, Princess Margaret Cancer Centre, Toronto, ON Canada; 5 School of Kinesiology, University of British Columbia, Vancouver, BC Canada

**Correction to: Journal of Behavioral Nutrition and Physical Activity (2018) 15:49**

**https://doi.org/10.1186/s12966-018-0686-0**

Following publication of the original article [1], the author has requested us to make a correction in the Results section of the Abstract and in the Discussion sections as explained below:

1. In the results section of the abstract, it should read ‘Overall adherence was 72% for total number of logins (i.e., > 3 visits each week)’ instead of ‘Overall adherence was 64% for total number of logins (i.e., > 3 visits each week)’.

2. In the discussion section of the abstract, it should read ‘Our study had an overall adherence rate of 72% that while higher than previous studies, still represents difficulties with engagement’ instead of ‘Our study had an overall adherence rate of 64% that while higher than previous studies, still represents difficulties with engagement.’
